# Association between systemic immune-inflammation index and serum neurofilament light chain: a population-based study from the NHANES (2013–2014)

**DOI:** 10.3389/fneur.2024.1432401

**Published:** 2024-08-22

**Authors:** Xinyu Liu, Yue Yang, Qiutong Lu, Jianshu Yang, Jing Yuan, Jun Hu, Yue Tu

**Affiliations:** ^1^Department of Traditional Chinese Medicine Rehabilitation, Acupuncture, Moxibustion and Massage College, Health Preservation and Rehabilitation College, Nanjing University of Chinese Medicine, Nanjing, China; ^2^Department of Big Data Management and Application, Health Economics and Management College, Nanjing University of Chinese Medicine, Nanjing, China; ^3^Department of Chinese Medicine, The First School of Clinical Medicine, Nanjing University of Chinese Medicine, Nanjing, China; ^4^Department of Acupuncture, Moxibustion and Massage, Acupuncture, Moxibustion and Massage College, Health Preservation and Rehabilitation College, Nanjing University of Chinese Medicine, Nanjing, China; ^5^Department of Traditional Chinese Medicine Health Preservation, Acupuncture, Moxibustion and Massage College, Health Preservation and Rehabilitation College, Nanjing University of Chinese Medicine, Nanjing, China

**Keywords:** systemic immune-inflammation index, serum neurofilament light chain, cross-sectional study, NHANES, neurological function

## Abstract

**Background:**

The systemic immune-inflammation index (SII) is a novel inflammatory marker used to assess the immune-inflammatory status of the human body. The systemic immune inflammation has an interplay and mutual relationship with neurological disorders. Serum neurofilament light chain (sNfL) is widely regarded as a potential biomarker for various neurological diseases. The study aimed to examine the association between SII and sNfL.

**Methods:**

This cross-sectional investigation was conducted in a population with complete data on SII and sNfL from the 2013–2014 National Health and Nutrition Examination Survey (NHANES). The SII was calculated by dividing the product of platelet count and neutrophil count by the lymphocyte count. Multivariate linear regression models and smooth curves were used to explore the linear connection between SII and sNfL. Sensitivity analyses, interaction tests, and diabetes subgroup smoothing curve fitting were also performed.

**Results:**

A total of 2,025 participants were included in our present research. SII showed a significant positive association with the natural logarithm-transformed sNfL (ln-sNfL) in crude model [0.17 (0.07, 0.28)], partially adjusted model [0.13 (0.03, 0.22)], and fully adjusted model [0.12 (0.02, 0.22)]. In all participants, the positive association between SII and ln-sNfL served as a linear relationship, as indicated by a smooth curve. Interaction tests showed that age, gender, BMI, hypertension, and diabetes did not have a significant impact on this positive association (*p* for interaction >0.05). The subgroup analysis of diabetes was conducted using smooth curve fitting. It was found that compared to the group without diabetes and the group in a pre-diabetic state, the effect was more pronounced in the group with diabetes.

**Conclusion:**

Our findings suggest that there is a positive association between SII and sNfL. Furthermore, in comparison to individuals without diabetes and those in a pre-diabetic state, the positive association between SII and sNfL was more pronounced in individuals with diabetes. Further large-scale prospective studies are needed to confirm the association between SII and sNfL.

## 1 Introduction

Neurofilament light chain (NfL) as a part of the neuronal structure, supports the radial growth of axons and maintains their size, shape, and caliber (1). When various factors lead to neuronal damage, NfL is released into the interstitial fluid and diffuses into the cerebrospinal fluid (CSF) and blood. For example, in the case of ischemic stroke (IS), a significant increase of NfL level can be detected in both CSF and blood (2). There is an association between serum NfL (sNfL) and CSF NfL concentration. Since peripheral blood is easier to collect, measuring serum level is considered as a more accessible and repeatable technique for assessing NfL level (3, 4). Currently, sNfL is widely recognized as a potential biomarker for various neurologic diseases (5). Recent studies have shown that NfL can be used as a biomarker to predict disease activity, severity, prognosis, and monitor treatment response in multiple sclerosis (MS) (6). A previous study conducted by Aamodt et al. has shown that Parkinson’s disease (PD) individuals with high plasma NfL are more likely to develop to cognitive impairment. Their results proved plasma NfL is a useful prognostic biomarker for PD, and predicted a clinical conversion to mild cognitive impairment or dementia (7). In addition to PD, NfL has also been shown to be a biomarker for various neurological diseases such as stroke, Alzheimer’s disease (AD), and others (8–10).

The systemic immune-inflammation index (SII) is a novel biomarker of inflammation that reflects the balance of inflammatory and immune status. The calculation method is as follows: SII = P × N/L, where P, N, and L represent the platelet, neutrophil, and lymphocyte counts, respectively. Initially developed by Hu et al., for predicting the prognosis of hepatocellular carcinoma patients (11), SII has subsequently been used in prognostic research for other tumors. SII has shown superior prognostic value compared to neutrophil–lymphocyte ratio (NLR), and platelet–lymphocyte ratio, making it a promising prognostic predictor for lung cancer (12). It has also been identified as an independent predictor of overall survival or progression free survival in gastrointestinal cancer patients (13), as well as being associated with the prognosis of breast cancer and colon cancer (14, 15). Furthermore, other related studies have suggested that SII can be used for prognostic research in inflammatory diseases such as cardiovascular and cerebrovascular diseases (16, 17).

In recent years, an increasing number of studies have found that inflammation affects the function of the neurological system. Axonal injury may play an important role in the pathogenesis of multiple system atrophy (MSA). Zhang et al. observed that blood biomarkers representing peripheral inflammation, such as C-reactive protein and NLR, are predictive biomarkers for wheelchair dependence in MSA patients. This supports the significance of inflammation in the prognosis of MSA (18). Brain injury biomarkers show an elevation in a severity-dependent manner during the acute phase of COVID-19, and these elevations are associated with both increased pro-inflammatory cytokines and the presence of autoantibodies, as well as with sNfL (19). Patients with acute ischemic stroke (AIS) typically present severe neurological disorder. Xu et al. found an association between SII and increased risk of overall stroke and its subtypes (20). Another study has explored the association between SII at admission and short and moderate term adverse outcomes in patients with AIS, which suggest that high SII is strongly associated with poor outcomes in stroke patients (17). Thus, SII may serve as an index for the progression of AIS in patients.

Previous study results indicated a close relationship between inflammation and the functioning of the nervous system. However, no studies have been reported on the association between SII and the neurologic disease biomarker sNfL. Therefore, our study aimed to clarify the relationship between SII and sNfL from a cross-sectional perspective, utilizing a large sample of data from the National Health and Nutrition Examination Survey (NHANES).

## 2 Materials and methods

### 2.1 Study population

In this cross-sectional study, we used publicly available data from the National Health and Nutrition Examination Survey (NHANES) from 2013 to 2014 conducted by the Centers for Disease Control and Prevention (CDC). NHANES is a series of cross-sectional, complex, multistage surveys conducted by the CDC to provide health and nutrition data on a nationally representative, non-institutionalized sample of the US population. More information can be found at: http://www.cdc.gov/nchs/nhanes/index.htm, which provides a detailed description of the NHANES survey’s continuous design. All study protocols in the NHANES survey protocol were authorized by the Ethics Review Board of the National Center for Health Statistics, and all participants signed informed permission.

**Figure 1:**
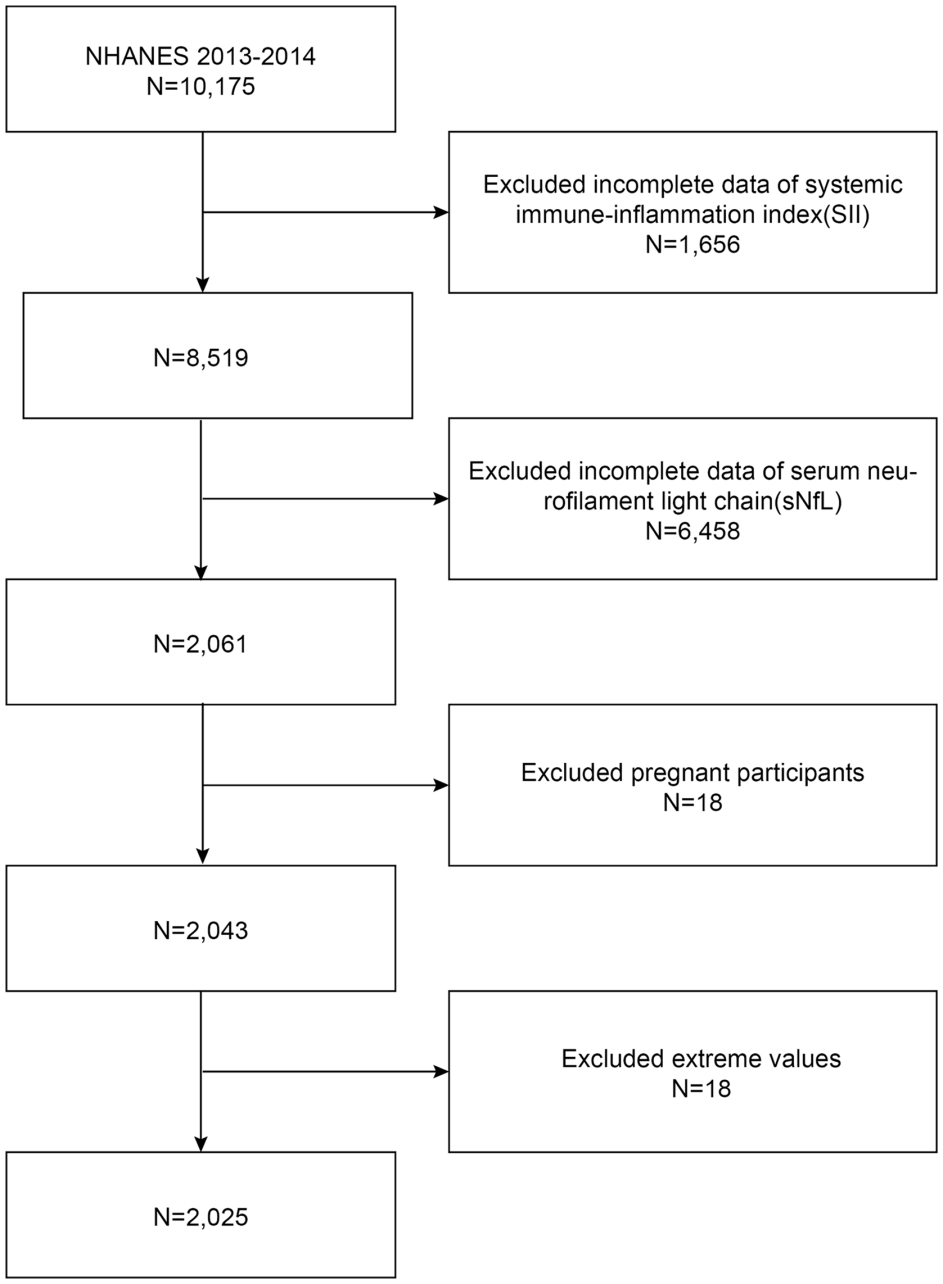
Flowchart of participant selection. NHANES, National Health and Nutrition Examination Survey.

Due to the limited availability of sNfL data, our analysis focused exclusively on the NHANES 2013–2014 cycle. The exclusion criteria for participants in our analysis were (1) missing complete data about SII (2) missing complete data about sNfL (3) pregnant, and (4) with extreme values of SII. A total of 10,175 participants were enrolled at first; after the exclusion of participants with missing data about SII (*n* = 1,656), sNfL (*n* = 6,458), those who were pregnant (*n* = 18), and those with extreme values of SII (*n* = 18), a total of 2,025 eligible participants aged ≥20 years were included in our final analysis (Figure 1).

### 2.2 Measurement of sNfL

The quantitative detection of sNfL utilized a highly sensitive immunoassay developed by Siemens Healthineers specifically for sNfL. This assay combined acridinium ester (AE) chemiluminescence and paramagnetic particle technology, enabling efficient integration into the fully automated Attelica immunoassay system. The method involved the formation of a stable antigen–antibody complex through specific binding of AE-labeled antibodies to the NfL antigen and capture antibodies coated on paramagnetic particles. Unbound antibodies were then accurately separated and removed, initiating a chemiluminescent reaction that is measured for light emission intensity, thereby achieving high-precision quantification of sNfL. To ensure the reliability and accuracy of analytical measurements, rigorous quality control and quality assurance procedures were implemented. This included regular analysis of low, medium, and high concentration quality control samples, as well as other replicate samples, with calculation of coefficients of variation and other relevant statistical data to describe the overall measurement range of the quality control samples. In practical applications, the lower limit of quantification (LLOQ) for this method was 3.9 pg/mL, while no cases exceeding the upper limit of quantification (ULOQ, 500 pg/mL) were observed in this study. Detailed information about the research methodology can be found on the relevant website.^1^

### 2.3 SII and covariates

SII served as the exposure variable in this investigation. Utilizing automated hematology analyzing devices (Coulter® DxH 800 analyzer), the lymphocyte, neutrophil, and platelet counts were measured and reported as ×10^3^ cells/μL. The SII level was determined by multiplying the platelet count by the neutrophil count divided by the lymphocyte count.

Based on previous researches, potential confounding factors linked with SII and sNfL were included in the final analysis. The covariates included age, gender, race, education level, income-to-poverty ratio, alcohol status, smoking status, BMI, waist circumference, hypertension, diabetes, hypercholesterolemia, stroke, and estimated glomerular filtration rate (eGFR). Among them, race was categorized as Mexican American, Non-Hispanic White, Non-Hispanic Black, other Hispanic, and other race. Education level was designated as less than high school, high school, and more than high school. Alcohol status was determined based on participants consuming at least 12 alcoholic drinks per year. Environmental tobacco smoke exposure is typically estimated using questionnaires, although questionnaires are not reliable. Studies have shown that cotinine is the optimal biomarker for nicotine exposure (21). Therefore, in this study, serum cotinine level was utilized as a measure of smoke exposure, and categorized into heavy smoke exposure (≥3 ng/mL), light smoke exposure (0.05 ~ 2.99 ng/mL), and no smoke exposure (< 0.05 ng/mL) groups based on previous research findings (22). BMI was categorized as <25, 25–29.9, and ≥ 30 kg/m^2^, corresponding to normal weight, overweight, and obesity, respectively. Diabetes was defined as a self-reported physician diagnosis of diabetes, use of insulin or oral hypoglycemic medication or having a hemoglobin A1c (HbA1c) level ≥ 6.5%, fasting plasma glucose (FPG) level ≥ 7 mmol/L, or 2 h oral glucose tolerance test (OGTT) plasma glucose level ≥ 11.1 mmol/L in accordance with the 2013 American Diabetes Association guidelines. Prediabetes was defined as any one of the following: 5.7% ≤ HbA1c < 6.5%, FPG between 5.6 mmol/L and 7.0 mmol/L, and a 2 h FPG value between 7.8 mmol/L and 11.1 mmol/L during an OGTT. Hypertension was diagnosed when at least one of the following criteria was fulfilled: systolic pressure/diastolic pressure ≥ 140/90 mmHg, a self-reported physician’s diagnosis of hypertension, or the self-reported use of hypertension medication. Similarly, hypercholesterolemia was confirmed if any of the following criteria were met: a cholesterol level ≥ 240 mg/dL, a self-reported physician’s diagnosis, or use of hypercholesterolemia medication. A history of stroke was determined if participants self-reported being previously diagnosed by a doctor. The eGFR was calculated using the Chronic Kidney Disease Epidemiology Collaboration (CKD-EPI) equation, which incorporates demographic factors such as gender, race, age, and serum creatinine to estimate the eGFR for each participant (23).

### 2.4 Statistical analysis

SII was divided into tertiles from lowest (T1) to highest (T3); continuous variables were expressed as means with standard deviations (SDs) and categorical variables as proportions. Because of the sNfL concentration value deviated from the normal distribution, we included the natural logarithm (ln) transformation of this variable in the analysis. The differences among participants grouped by SII tertiles were assessed using a weighted t-test. To examine the association between SII and the natural logarithm-transformed sNfL (ln-sNfL), multiple linear regression analysis between SII and ln-sNfL was used to construct multivariate tests using three models: model 1: no variables adjusted; model 2: gender, age, and race adjusted; model 3: adjusted for all covariates. And SII and ln-sNfL were evaluated using standardized regression coefficient (β) and 95% confidence interval (CI) in the models. In sensitivity analysis, linear trend tests were conducted with SII tertile groups as independent variables to evaluate its robustness. Using three models, multivariate tests were constructed by controlling for variables and fitting a smooth curve. Additionally, subgroup analysis and interaction tests were conducted to explore the relationship between SII and ln-sNfL in different groups. Finally, a smooth curve was fitted to the subgroup of diabetes. The statistical analyses were conducted using R studio (Version 4.2.2) and EmpowerStats (Version 2.0). A *p*-value <0.05 was determined to be significant. In order to mitigate the significant volatility of our dataset, we employed a weighting approach.

## 3 Results

### 3.1 Baseline characteristics of participants

A total of 2,025 participants were involved, with an average age of 45.14 ± 15.14 years and a gender split of 49.29% male to 50.71% female. The quartiles for SII and sNfL are as follows: for SII, the first quartile (Q1) is 309.93, the second quartile (Q2) is 423.69, and the third quartile (Q3) is 595.65; for sNfL, the first quartile (Q1) is 8.2 pg/mL, the second quartile (Q2) is 12.3 pg/mL, and the third quartile (Q3) is 19.1 pg/mL.

Participants were divided into three groups based on the tertiles of SII: T1 group (*n* = 675), T2 group (*n* = 675), and T3 group (*n* = 675). The clinical characteristics of the participants according to SII as a column-stratified variable are shown in Table 1. SII was statistically significant with age, gender, race, education level, serum cotinine level, BMI, waist circumference, diabetes, hypertension, hypercholesterolemia, and sNfL (*p* < 0.05). Compared to those with low SII, individuals with high SII tended to be older, female, non-Hispanic white or Mexican American, with a BMI ≥ 30 kg/m^2^, larger waist circumference, lower education level, higher serum cotinine level, and had higher prevalence of diabetes, hypertension, hyperlipidemia, as well as having higher level of sNfL.

**Table 1:** Baseline characteristics of study population according to SII tertiles, weighted.

SII	Overall	T1 group (*n* = 675)	T2 group (*n* = 675)	T3 group (*n* = 675)	*p* for trend
**Age (years)**	45.14 ± 15.14	43.81 ± 15.55	45.78 ± 14.81	45.70 ± 15.00	0.0294
**Gender (%)**					<0.0001
Male	49.29	55.89	52.29	40.57	
Female	50.71	44.11	47.71	59.43	
**Race (%)**					<0.0001
Mexican American	9.62	9.44	9.60	9.81	
Other Hispanic	5.84	4.73	6.48	6.22	
Non-Hispanic White	65.17	59.50	66.05	69.34	
Non-Hispanic Black	11.67	17.83	9.04	8.73	
Other Races	7.70	8.49	8.83	5.91	
**Education level (%)**					0.0219
Less than high school	15.84	16.92	12.76	17.83	
High school	20.19	21.59	18.55	20.53	
Above high school	63.97	61.50	68.69	61.64	
**Income-to-poverty ratio**	2.91 ± 1.69	2.98 ± 1.71	2.95 ± 1.69	2.80 ± 1.67	0.1231
**Serum cotinine level (%)**					0.0140
< 0.05 ng/mL	55.28	57.81	57.72	50.70	
0.05–2.99 ng/mL	16.27	17.16	15.04	16.67	
≥ 3 ng/mL	28.45	25.03	27.24	32.63	
**Alcohol status (%)**					0.0628
Yes	78.72	75.45	79.94	80.45	
No	21.28	24.55	20.06	19.55	
**BMI (%)**					<0.0001
<25	29.49	31.78	29.87	27.08	
25–30	32.52	36.73	33.82	27.55	
≥30	37.99	31.49	36.31	45.36	
**Waist circumference (cm)**	99.76 ± 17.12	97.04 ± 15.56	99.53 ± 15.85	102.48 ± 19.13	<0.0001
**eGFR (ml/min/1.73 m^2^)**	95.99 ± 20.57	96.84 ± 19.87	95.78 ± 20.27	95.44 ± 21.42	0.4366
**Diabetes (%)**					0.0024
No	49.17	54.73	48.09	45.28	
Yes	13.66	10.60	13.48	16.54	
Prediabetes	37.17	34.66	38.43	38.19	
**Hypertension (%)**	36.91	33.38	35.84	41.07	0.0112
**Hypercholesterolemia (%)**	40.22	34.40	42.71	42.98	0.0016
**Stroke (%)**	2.47	2.24	2.65	2.49	0.8872
**ln-sNfL**	2.54 ± 0.67	2.48 ± 0.60	2.53 ± 0.64	2.60 ± 0.74	0.0034

Mean ± SD for continuous variables: the *p*-value was calculated by weighted linear regression model. % for categorical variables: the *p*-value was calculated by a weighted chi-square test. BMI, body mass index; eGFR, estimated glomerular filtration rate; SII, systemic immune-inflammation index; ln-sNfL, the natural logarithm-transformed serum neurofilament light chain.

### 3.2 Association between SII and ln-sNfL

**Figure 2:**
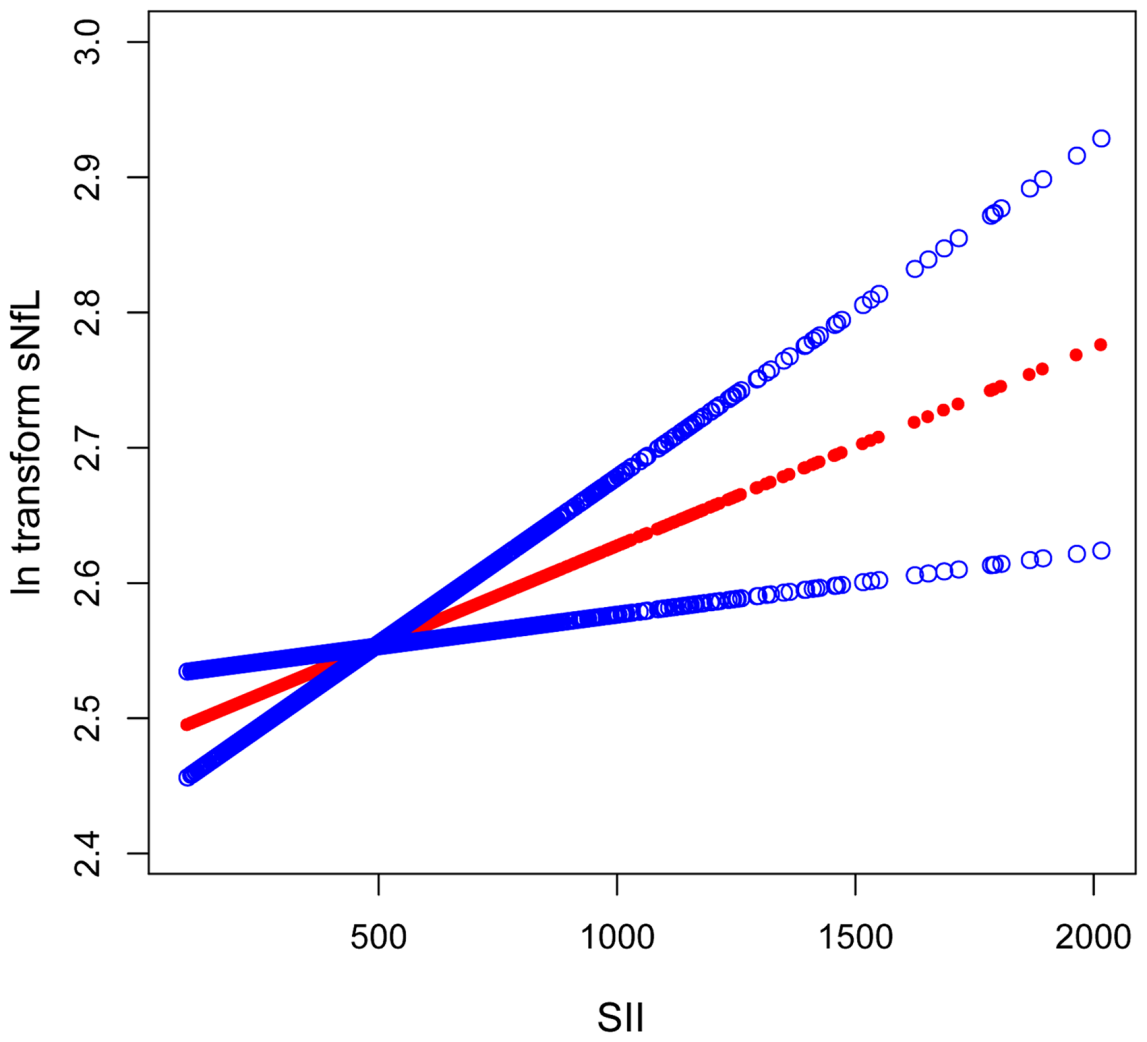
The association between SII and ln-sNfL. The solid red line represents the smooth curve fit between variables. Blue bands represent the 95% confidence interval from the fit. SII, systemic immune-inflammation index; ln-sNfL, the natural logarithm-transformed serum neurofilament light chain.

Because the effect value was not apparent, SII/1000 was used to amplify the effect value by 1,000 times. The constructed weighted multivariate linear regression models are listed in Table 2. SII showed a significant positive association with sNfL in the crude model, the partially adjusted model, and the fully adjusted model [model 1: 0.17 (0.07, 0.28); model 2: 0.13 (0.03, 0.22); model 3: 0.12 (0.02, 0.22)]. In the fully adjusted model, each one-unit increase in SII/1000 score was associated with a 0.12-unit increase in ln-sNfL. The results of the trend test further demonstrated that the above linear relationships remained stable across SII tertiles (*p* for trend < 0.05), with ln-sNfL in T3 group of SII being 0.10-unit increase than those in T1 group under the same conditions. Smooth curve was also performed, which showed a linear positive association between SII and ln-sNfL (Figure 2).

**Table 2:** The association between SII and ln-sNfL.

	Crude Model (Model 1) β (95%CI), *p*-value	Partially Adjusted Model (Model 2) β (95%CI), *p*-value	Fully Adjusted Model (Model 3) β (95%CI), *p*-value
SII/1000	0.17 (0.07, 0.28) 0.0016**	0.13 (0.03, 0.22) 0.0079**	0.12 (0.02, 0.22) 0.0166*
Categories			
T1 group	Reference	Reference	Reference
T2 group	0.05 (−0.02, 0.13) 0.1474	0.01 (−0.05, 0.08) 0.6656	0.02 (−0.05, 0.08) 0.5898
T3 group	0.12 (0.05, 0.19) 0.0008**	0.10 (0.03, 0.16) 0.0023**	0.10 (0.03, 0.17) 0.0031**

Model 1, no covariates were adjusted. Model 2, age, gender, and race were adjusted. Model 3, age, gender, race, education level, income-to-poverty ratio, alcohol status, smoking status, BMI, waist circumference, hypertension, diabetes, hypercholesterolemia, stroke, and eGFR were adjusted. 95% CI, 95% confidence interval; β, standardized coefficient; SII, systemic immune-inflammation index; ln-sNfL, natural logarithm transformation of serum neurofilament light chain. *p < 0.05, **p < 0.01; *p* < 0.05 was considered statistically significant.

### 3.3 Subgroup analysis

**Figure 3:**
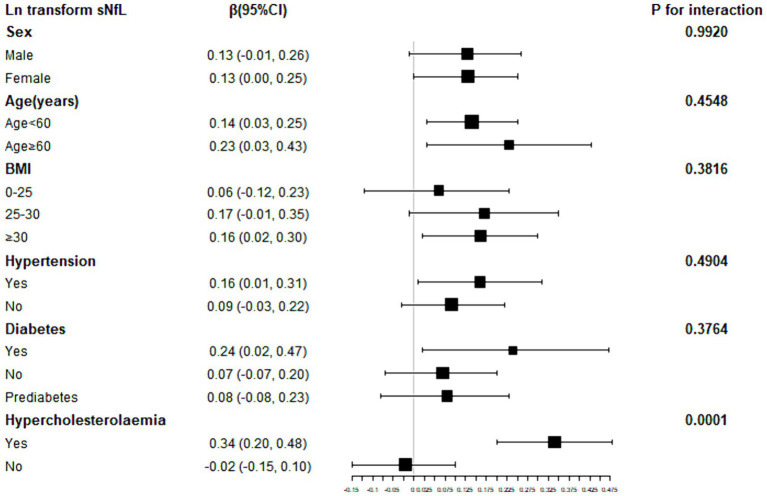
Subgroup analysis for the association between SII and ln-sNfL. SII, systemic immune-inflammation index; ln-sNfL, the natural logarithm-transformed serum neurofilament light chain.

To further explore factors influencing the association between SII and sNfL, we conducted stratified analyses based on sex, age, BMI, hypertension, diabetes, and hypercholesterolemia. Further subgroup analyses revealed that the association between SII and ln-sNfL was not consistent, as shown in Figure 3. Significant associations between SII and ln-sNfL (*p* < 0.05) were observed across all age groups, including females and individuals with obesity, hypertension, diabetes, and hypercholesterolemia. Interaction tests indicated that sex, age, BMI, hypertension, and diabetes did not significantly influence this positive association (*p* for interaction >0.05).

**Figure 4:**
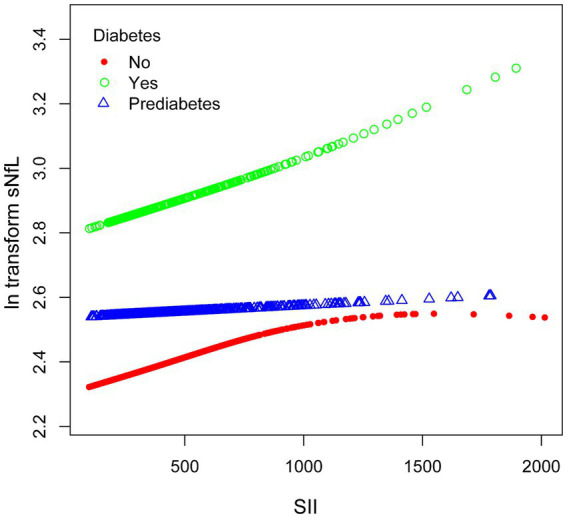
The association between SII and ln-sNfL stratified by diabetes. SII, systemic immune-inflammation index; ln-sNfL, the natural logarithm-transformed serum neurofilament light chain.

We performed subgroup smooth curve fitting for individuals with diabetes (Figure 4), and the results indicated that the effect size was more significant in individuals with diabetes compared to those without diabetes and those in a pre-diabetic state.

## 4 Discussion

In our cross-sectional study, we found an association between higher SII values and higher sNfL concentrations. The results from subgroup analysis and interaction tests indicated that this association was consistent across the population. In the subgroup analysis, we also observed that the effect was more pronounced in individuals with diabetes compared to those without diabetes and those in a prediabetic state.

As far as we know, no previous research has investigated the association between SII and sNfL. Previous studies have reported the relationship between inflammation and NfL. For instance, Disanto et al. found a significant positive association between sNfL and focal inflammatory MRI lesions in both brain and spinal cord (24). Their analysis of clinical variables associated with sNfL showed that in addition to age, the presence of recent relapses and disability as measured by the Extended Disability Status Scale (EDSS) were independently and positively associated with sNfL level. The results suggest that sNfL level may be associated with acute inflammatory injury and chronic diffuse neuronal loss. Li et al. discovered NfL level notably increased in anti-N-methyl-d-aspartate receptor (NMDAR) encephalitis patients in acute phase and positively correlated with disease severity (25). In anti-NMDAR encephalitis patients, NfL was positively correlated with pro-inflammatory cytokines and modified Rankin Scale (mRS) scores, suggesting that NfL may be associated with inflammatory responses. Moreover, interleukin (IL)-1β is considered as a contributing factor in autoimmune inflammatory diseases (26). Meyer et al. demonstrated an association between elevated level of inflammatory cytokines (IL-6 and IL-5) and NfL (27). Additionally, studies utilizing high-throughput and scalable assays have shown that sNfL is associated with clinical disability, inflammatory disease activity, and whole-brain atrophy in MS (28). Consistent with most studies, our research indicates a positive association between SII and sNfL level, suggesting that high SII may independently cause or worsen neurological disorders.

Currently, sNfL has been widely considered as a potential biomarker for neurological diseases (5). Previous research has found that elevated level of NfL in CSF and serum are often associated with clinical progression in primary neurodegenerative diseases (29). An investigation based on an ultrasensitive sNfL assay in healthy controls and independent MS patients found that sNfL level in MS patients were not only significantly elevated, but also correlated with the presence and activity of focal lesions in the brain and spinal cord, confirming the value of sNfL level as a biomarker of tissue damage in MS (5). In a cross-sectional study, elevated sNfL level was found to be positively correlated with an increased risk of early PD-related symptoms, and that sNfL level was significantly negatively correlated with cognitive function test results, suggesting that sNfL may serve as a potential biomarker for early PD (24). Additionally, a previous study found that elevated sNfL level in the late phase after stroke can be used as a biomarker for adaptive neural plasticity and a positive predictor of functional improvement, indicating that sNfL can predict the adverse outcome in the acute phase after stroke and the improvement in the late phase (8). Furthermore, Steffen Tiedt et al. found that after adjusting for age, gender, hypertension and recurrent ischemic lesions, the association between sNfL level and secondary neurodegeneration still existed, indicating that sNfL is expected to be a biomarker for monitoring primary and secondary axonal injury and predicting functional outcomes after IS (30). All in all, various studies have shown the wide application of sNfL in clinical aspects.

The underlying mechanism of this positive association between SII and sNfL has not been well understood. We speculate that the increased SII level in neuroinflammation and the related inflammatory factors disrupt blood–brain barrier (BBB) to induce neurodegeneration and worsen the damage of the nervous system, thus causing an increase in NfL level in the CSF. Research has shown that the main factor affecting the structure and function of BBB is inflammation, which is mainly caused by cytokines secreted by immune cells including IL-1β, IL-6 and tumor necrosis factor (TNF)-α (31). For instance, a rapid activation of proinflammatory cytokines such as IL-1β, IL-6 and TNF-α was observed both in animal models of acquired epilepsy and in brain tissue obtained from patients with temporal lobe epilepsy or cortical developmental malformations undergoing epilepsy surgery (32). Additionally, other cytokines have been shown to play a destructive role in BBB during neuroinflammation. Hania Kebir et al. demonstrated the expressions of IL-17 and IL-22 receptors on the BBB endothelial cells within MS lesions, thus indicating their involvement in disrupting the tight junctions (TJs) of BBB (33). Yosef N et al. demonstrated that IL-17 can induce the release of inflammatory factors causing tissue infiltration and destruction, promote the maturation and chemotactic of dendritic cells and stimulate the activation of T cells (34). These findings suggest that the disruption of the BBB by inflammatory cytokines may contribute to the observed association between SII and sNfL. However, further research is needed to fully understand the complex relationship between systemic inflammation, neuroinflammation, BBB integrity, and neurodegeneration. These cytokines, which destroy BBB during neurodegeneration, may provide some promising new targets for clinical applications to treat neurological diseases and injuries. In the process of neurodegeneration and neuroinflammation, relevant inflammatory cell cytokines integrate with their specific receptors to disrupt TJs and transendothelial electrical resistance of the BBB (35, 36). In addition, aging makes BBB more vulnerable to the destruction of inflammatory cytokines, and the damage of BBB is easy to recruit more immune cells and cytokines into the brain parenchyma, thus inducing neurodegeneration (37). A study found that BBB structure can be damaged with age, making patients susceptible to neurological diseases such as AD (38). In summary, for the treatment of neurological diseases, it can be considered to develop new clinical therapies by controlling inflammation and regulating the major cytokines attacking BBB. Therefore, it is necessary to study the relationship between SII and sNfL.

In the subgroup analysis, the different states of diabetes and hyperlipidemia significantly influenced the association pattern were observed between SII and ln-sNfL. When exploring the impact of hyperlipidemia on this relationship, a smooth curve was attempted to be fit, similar to the approach for diabetes analysis using in this study. Unfortunately, due to the interference of extreme values in the SII data, the stable and highly credible conclusion was not able to be drawn. Therefore, this part of the findings was not included in the results. Instead, the focus was shifted to the crucial factor of diabetes, and it was found that higher sNfL levels were exhibited by diabetics compared to those without diabetes or participants in a pre-diabetic state. Furthermore, the positive association between SII and sNfL appeared to be more phanerous in the diabetes. However, the underlying mechanisms behind this relationship have not been well elucidated. We speculate on the following reasons: the state of hyperglycemia may lead to chronic inflammation, oxidative stress and endothelial cell dysfunction, which in turn can contribute to reduced kidney function (39, 40). This reduced kidney function may result in decreasing renal clearance and glomerular filtration rate of NfL, while also reducing the synthesis of neuroprotective substances produced by the kidneys, such as erythropoietin and vitamin D, which could potentially lead to neuronal damage (41–43). Additionally, the obesity associated with hyperglycemia and proinflammatory metabolism produced by insulin resistance (44) may further exacerbate neuronal and vascular damage (45, 46), neurotoxicity, and changes in osmotic pressure, ultimately causing damage to BBB (47) and resulting in elevated sNfL level. The consequences of the proinflammatory metabolism can lead to endothelial cell dysfunction (48–50), which in turn may contribute to or exacerbate insulin resistance (51). Hyperglycaemia can also result in thickening the capillary basement membrane, increasing endothelial permeability, and inducing dysfunction of endothelial and vascular smooth muscle cells. These damages further promote the effect of the inflammatory response on sNfL level. Moreover, hyperglycaemia can cause demyelination and axonal loss in peripheral sensation and motor nerves, leading to the release of NfL into the bloodstream. Glycemic fluctuations in acute diabetes may also activate microglial, thereby exacerbating oxidative stress in the body and causing neuronal damage, resulting in increased level of sNfL (52).

This study has several strengths. Firstly, to our knowledge, this is the first study to examine the association between SII and sNfL. Secondly, the large sample size and the standardized protocols of the NHANES not only minimize potential biases but also enhance the reliability of the results. Additionally, we adjusted for relevant covariates to ensure the generalizability of our findings to a broad population, thereby improving the validity and representativeness of this study. However, this study also has limitations. The cross-sectional study design does not allow us to determine causality, and extensive prospective research is needed to elucidate any causal relationships. While we controlled for certain confounding factors, other confounders may still have an impact on the outcomes.

## 5 Conclusion

Our results indicated a positive association between SII and sNfL. In subgroup analysis, significant association between SII and sNfL was observed consistently across all age groups, including females and individuals with obesity, hypertension, diabetes, and hypercholesterolemia (*p* < 0.05). Furthermore, the positive association between SII and sNfL was more pronounced in individuals with diabetes compared to those without diabetes or in a pre-diabetic state. However, these findings do not establish a causal relationship and more studies are expected.

## Data Availability

The datasets presented in this study can be found in online repositories. The names of the repository/repositories and accession number(s) can be found at: https://www.cdc.gov/nchs/nhanes/.

## Footnotes

^1^https://wwwn.cdc.gov/Nchs/Nhanes/2013-2014/SSSNFL_H.htm
